# Prevalence of Bisphosphonate and Denosumab Use in Elderly Care Facilities: Implications for the Management of Medication-Related Osteonecrosis of the Jaw

**DOI:** 10.7759/cureus.83490

**Published:** 2025-05-05

**Authors:** Motohiko Sano, Yosuke Iijima, Miki Yamada, Mihoko Kanbe, Tomoyuki Kanbe, Shunsuke Hino, Kiyoko Ariya, Norio Horie, Takahiro Kaneko

**Affiliations:** 1 Division of Applied Pharmaceutical Education and Research, Hoshi University, Tokyo, JPN; 2 Department of Oral and Maxillofacial Surgery, Saitama Medical Center, Saitama Medical University, Saitama, JPN; 3 Department of Oral and Maxillofacial Surgery, Saitama Yorii Hospital, Saitama, JPN; 4 Special Elderly Care Home, Attaka No ie, Saitama, JPN

**Keywords:** bisphosphonate-related osteonecrosis of the jaw, elderly inpatient, intravenous bisphosphonate, pharmacist collaboration, pharmacist role

## Abstract

Objective: Our group is investigating the contribution of pharmacists in reducing medication-related osteonecrosis of the jaw (MRONJ) associated with bisphosphonates (BPs) and denosumab (Dmab). Recently, our group encountered a case of MRONJ occurring in an elderly care setting. The aim of this study was therefore to investigate the actual use of BP and Dmab and the incidence of MRONJ in elderly care facilities. We also discussed measures for the prevention and early detection of MRONJ by dentists and pharmacists associated with these facilities.

Materials and methods: A cross-sectional survey across four elderly care facilities (including the one where the case occurred) was conducted to determine the prevalence of BP and Dmab use and related factors, as well as the incidence of MRONJ.

Results: Among 327 residents, 9.8% (32) were receiving BP or Dmab therapy (84.4% oral, 15.6% injectable). The encountered MRONJ case was the only one identified, occurring in a resident using injectable BP.

Conclusion: In these elderly facilities, 9.8% of residents used BPs or Dmab, with 15.6% receiving injectable formulations. One MRONJ case occurred with injectable BP use, suggesting the potential for sporadic MRONJ in elderly care. As BP/Dmab use is likely to increase, pharmacist intervention for prescription review and training for visiting dentists are considered effective strategies for MRONJ prevention and early detection.

## Introduction

Antiresorptive medications, bisphosphonates (BPs) and denosumab (Dmab), are widely used for osteoporosis treatment and the management of skeletal-related events in cancer patients [1-3]. While effective in reducing bone resorption and increasing bone density, these drugs are associated with adverse events, notably medication-related osteonecrosis of the jaw (MRONJ) [4,5].

The pathogenesis of MRONJ is not fully elucidated, but it is hypothesized that BPs and Dmab inhibit osteoclast activity, suppressing bone remodelling and causing osteocyte expiration and necrosis without replacement by new bone, eventually leading to jaw osteonecrosis [6,7]. Inflammatory cytokines and bacterial infections also contribute to MRONJ development, highlighting the importance of oral hygiene [8-13].

The risk of MRONJ is reported to be 0.02%-0.05% for BPs and 0.3% for Dmab in osteoporosis, with higher risks in cancer treatment (up to 5% or higher depending on the regimen and duration) [4]. Although the incidence of MRONJ in osteoporosis is relatively low, it is a refractory condition that, in advanced stages, requires marginal or segmental resection of the jawbone, which impairs feeding function and significantly interferes with patient quality of life [14-16].

Given the increasing prevalence of osteoporosis treatment and the expanding use of BPs and Dmab in cancer management, the number of patients susceptible to MRONJ is expected to rise. While MRONJ is well-recognized among dentists and oral surgeons, awareness remains limited among other healthcare professionals, including pharmacists, who do not directly administer these medications. Our research group, focused on enhancing pharmacist involvement in MRONJ risk reduction [17,18], recently encountered a notable case of MRONJ in an osteoporotic resident of an elderly care facility.

Notably, there is a paucity of data regarding BP and Dmab administration and MRONJ incidence within elderly care settings, environments where access to advanced medical information can be limited. This observation, coupled with our team's experience of MRONJ in such a setting, prompted this investigation. The primary objectives of this study were twofold: first, to report the prevalence of BP and Dmab use and the occurrence of MRONJ within elderly care facilities; and second, to discuss the potential risk factors for MRONJ development in this specific setting and explore the role of interprofessional collaboration, particularly involving pharmacists and visiting dentists, in its prevention and early detection.

## Materials and methods

**Figure 1:**
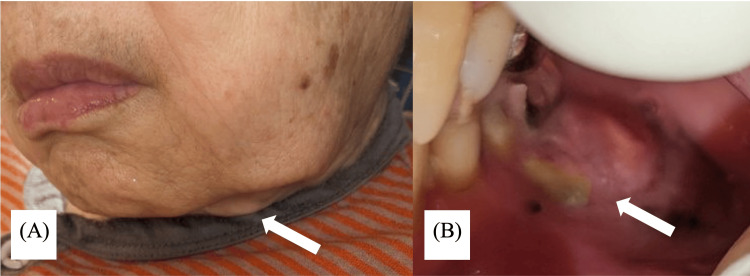
Photographs taken when symptoms were first noticed. (A) Painless swelling is noted along the inferior border of the mandible on the buccal side of the left mandibular first molar. (B) Intraorally, bone exposure is seen on the buccal side of the left lower first molar.

**Figure 2:**
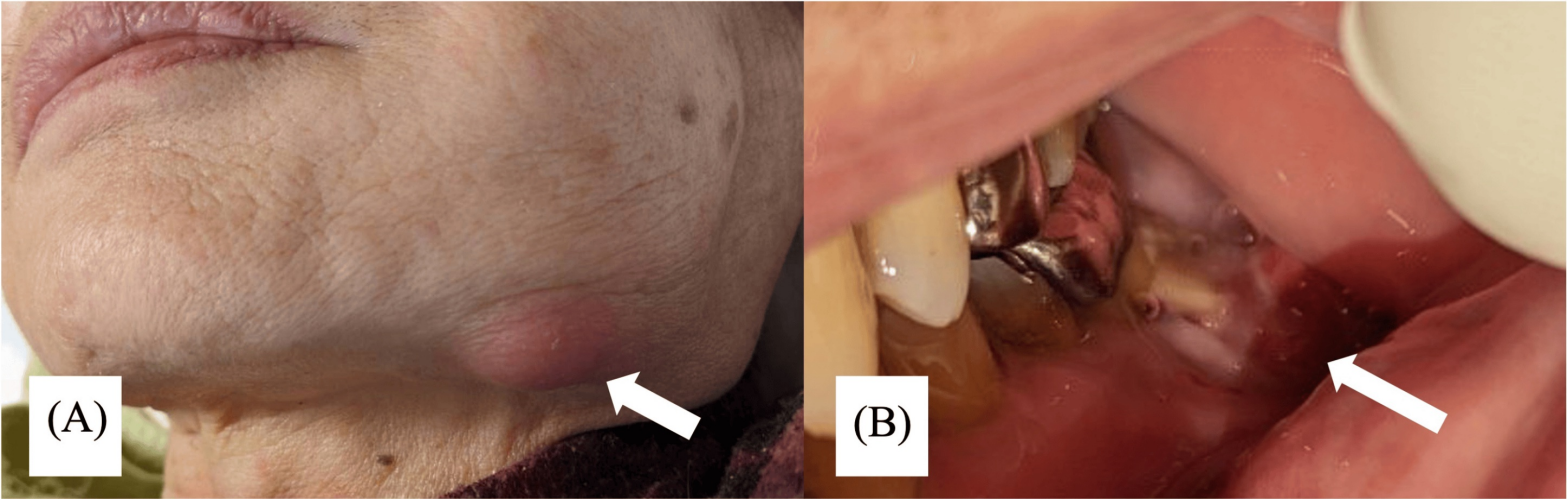
Photographs one week later (after starting antibiotics). (A) An abscess is seen on the inferior margin of the left mandible. (B) Intraorally, bone exposure is more evident.

An 82-year-old female resident presented with left mandibular swelling detected during routine oral care by an oral hygienist. Intraoral examination revealed exposed bone on the buccal aspect of the left lower first molar (Figures 1A, 1B, 2A, 2B). Her medical history included osteoporosis treated with annual intravenous zoledronic acid. The visiting dentist initially did not suspect MRONJ due to the lack of oral BP history and recent extractions. This case highlighted diagnostic challenges related to injectable BP administration and non-extraction-related MRONJ.

This retrospective observational study, including a background investigation of BP and Dmab use and the described case report, was conducted in accordance with the ethical principles outlined in the Declaration of Helsinki and Good Clinical Practice guidelines. The study protocol was approved by the Ethics Committee of Saitama Medical University (approval no. 2024-038). As this was a retrospective study, information regarding the research protocol was posted on the Ethics Committee website to ensure transparency and provide a point of contact for any inquiries from subjects. The background investigation involved residents of four elderly care facilities (special nursing homes, designated as Facilities A, B, C, and D) where oral surgeons from the Department of Oral and Maxillofacial Surgery, Saitama Medical Center, Saitama Medical University, provide regular consultations for oral surgery-related conditions. These facilities include the one facility where the MRONJ reported here developed. Data were collected from all residents who were present in the facilities as of October 21, 2024. The following data were extracted from the residents' medical records: Demographic information (age and sex), BP and Dmab usage (number of users, indications for use, routes of administration (oral or injectable), and the occurrence of MRONJ. Data extraction was performed by nurses responsible for managing the residents' health records at each facility.

The diagnosis of MRONJ was made according to the diagnostic criteria of the American Association of Oral and Maxillofacial Surgeons (AAOMS), which are defined as follows: 1) Current or previous treatment with antiresorptive therapy alone or in combination with immune modulators or antiangiogenic medications. 2) Exposed bone or bone that can be probed through an intraoral or extraoral fistula(e) in the maxillofacial region that has persisted for more than eight weeks. 3) No history of radiation therapy to the jaws or metastatic disease to the jaws. The diagnosis of MRONJ was made by the oral surgeon on the basis of history, medication history, clinical symptoms, including radiographic examination.

The data collected were tabulated for each facility on a Microsoft Excel sheet (Microsoft Corporation, Washington, DC) and processed as a whole. This study aimed to investigate the prevalence of BP and Dmab use among residents of Facilities A to D. Due to the limited sample size and the observed homogeneity of results in certain facilities, formal statistical analyses were not performed. Instead, descriptive statistics were utilized to summarize the data.

## Results

A total of 327 residents across four elderly care facilities were included in the background investigation: 100 from Facility A, 53 from Facility B, 90 from Facility C, and 84 from Facility D (Table 1).

**Table 1:** Details of bisphosphonate (BP) and denosumab (Dmab) use in elderly care facilities. *, Injection of bisphosphonate. MRONJ, medication-related osteonecrosis of the jaw.

	Facility A	Facility B	Facility C	Facility D	Total
	n = 100	n = 53	n = 90	n = 84	n = 327
Age (year)					
Mean± SD	86.9 ± 6.6	87.9 ± 7.2	87.7 ± 8.2	85.7 ± 9.2	87.0 ± 7.8
Sex					
Male	29/100 (29.0%)	8/53 (15.1%)	29/90 (32.2%)	24/84 (28.6%)	90/327 (27.5%)
Female	71/100 (71.0%)	45/53 (84.9%)	61/90 (67.7%)	60/84 (71.4%)	237/327 (72.5%)
BPs or Dmab use					
Yes	14/100 (14.0%)	9/53 (7.0%)	3/90 (3.3%)	6/84 (7.1%)	32/327 (9.8%)
No	86/100 (86.0%)	44/53 (83.0%)	87/90 (96.7%)	78/84 (92.9%)	295/327 (90.2%)
Reason for administration					
Osteoporosis	14/14 (100.0%)	9/9 (100.0%)	3/3 (100.0%)	6/6 (100%)	32/32 (100.0%)
Cancer	0/14(0.0%)	0/9 (0.0%)	0/3 (0.0%)	0/6 (0.0%)	0/32 (0.0%)
Route of administration					
Oral	11/14 (78.6%)	8/9 (88.9%)	2/3 (66.7%)	6/6 (100%)	27/32(84.4%)
Injection	3/14 (21.4%)	1/9 (11.1%)	1/3 (33.3%)	0/6 (0.0%)	5/32 (15.6%)
Type of injectable drug					
BPs	2/3 (66.7%)	1/1 (100.0%)	0/1 (0.0%)	0/0 (0.0%)	3/5 (60.0%)
Dmab	1/3 (33.3%)	0/1 (0.0%)	1/1 (100.0%)	0/0 (0.0%)	2/5 (40.0%)
Onset of MRONJ	0/14 (0.0%)	1/9 (11.1%)*	0/3 (0.0%)	0/6 (0.0%)	1/32 (3.1%)

The overall, the mean age ± standard deviation of the residents was 87.0 ± 7.8 years, 90 (27.5%) were male and 237 (72.5%) were female, with a male to female ratio of 1:2.6. BPs and Dmab usage were identified in 9.8% (32/327) of the residents. All BP and Dmab prescriptions were for the treatment of osteoporosis; no residents were receiving these medications for cancer-related indications. The routes of administration were oral in 84.4% (27/32) of cases and injectable in 15.6% (5/32) of cases. Of the injectable administrations, three were BPs, and two were Dmab. One case of MRONJ was identified in Facility B (BP injection), which is the case briefly reported in the Materials and Methods section and will be further discussed. The MRONJ incidence among BP/Dmab users in this investigation was 3.1% (1/32).

## Discussion

This study identifies the prevalence of BP and Dmab use in elderly care facility residents and discusses crucial measures for MRONJ prevention and early detection, highlighting the roles of both visiting dentists and outsourced pharmacists. The inclusion of a specific MRONJ case underscores the clinical relevance of these findings within this vulnerable population.

Notably, there is a paucity of data regarding BP and Dmab administration and MRONJ incidence within elderly care settings. This observation prompted an investigation into the prevalence of BP and Dmab use within these facilities to better understand the potential risk and inform preventative strategies. Elderly care facilities primarily focus on providing care to older adults; however, facility staff and caregivers may lack comprehensive medical literacy. Therefore, healthcare professionals must proactively disseminate essential medical information. The occurrence of MRONJ in elderly care facilities warrants increased attention.

Medications administered for various conditions can impact oral health and dental treatment outcomes [19,20]. MRONJ, caused by BPs and Dmab, commonly used in the treatment of osteoporosis and cancers prone to bone metastasis, is a significant drug-related dental disease. Osteoporosis is a substantial health concern in Japan, affecting an estimated 13-15 million people. The prevalence of osteoporosis increases with age, reaching approximately 30%-50% in women in their 70s and 40%-90% in those in their 80s, depending on the measurement site. It is estimated that only 20% of osteoporosis patients receive treatment [21-23]. Our background investigation revealed that 9.8% of residents used BPs/Dmab solely for osteoporosis, suggesting potential undertreatment in elderly facilities. With increasing BP/Dmab use for fracture prevention and cancer, MRONJ prevention, and oral care for affected patients will be increasingly important for facility-affiliated dentists [14].

While the observed MRONJ incidence of 3.1% among BP/Dmab users appears notably higher than reported in general osteoporosis populations (0.02%-0.05% for BPs and 0.3% for Dmab) [4], the small sample size and the study's initiation following the identification of an MRONJ case necessitate a cautious interpretation of this specific rate. This finding serves as a critical reminder of the potential for sporadic MRONJ occurrences in elderly facilities. The presented case further emphasizes diagnostic challenges related to less common presentations, such as injectable BP use and non-extraction-related onset.

Effective MRONJ prevention and early detection require dentists to accurately diagnose the condition. The reported case highlights diagnostic pitfalls, particularly the initial lack of information regarding injectable drug use. Despite routine medical history reviews, the rising prevalence of injectable BPs/Dmab, favored for adherence and tolerability [24], may lead to overlooked risk factors. Our investigation found 15.6% injectable BP/Dmab use in these facilities. While oral BPs are generally first-line, injectable formulations offer convenience and are likely to increase in elderly care.

Furthermore, the MRONJ case originating from periodontal disease, not extraction, underscores the need for dentists to recognize diverse MRONJ etiologies [25-28]. Enhancing MRONJ knowledge among visiting dentists, enabling them to consider it in cases of unexplained inflammation or bone exposure, is crucial. Sharing information on at-risk residents with dental hygienists, who are often the first to observe oral changes, is also essential.

Based on our experience, existing literature [14,29], and the challenges highlighted by the reported case, we suggest that pharmacist-led prescription reviews and educational initiatives for visiting dentists could be valuable components of a comprehensive MRONJ prevention strategy in elderly care facilities. However, further research is needed to empirically evaluate the effectiveness of these interventions in this specific setting.

While the primary focus of this study was MRONJ, the broader involvement of pharmacists as drug specialists has the potential to improve overall dental care for elderly residents on multiple medications. The lack of awareness among facility staff regarding the osteoporosis medication-MRONJ link emphasizes the urgent need for proactive education by healthcare professionals. Addressing MRONJ effectively requires a comprehensive understanding of contributing factors within the facility setting.

The primary limitation of this study is the small sample size of the background investigation across four facilities, which restricts the generalizability of the BP and Dmab usage and MRONJ incidence rates. Larger-scale studies across more diverse elderly care settings are warranted to obtain more precise epidemiological data and to identify specific risk factors within this population. Furthermore, there is a potential for reporting bias or underdiagnosis of MRONJ. This could occur due to the subtle initial symptoms of MRONJ being overlooked, a lack of comprehensive MRONJ knowledge among non-dental healthcare professionals in the facilities, or insufficient communication between medical and dental staff. Such bias might lead to an underestimation of the true MRONJ prevalence in these settings. The diagnosis of MRONJ in this study was made by the oral surgeon based on clinical and radiographic findings, by the AAOMS criteria. However, the absence of a standardized diagnostic confirmation protocol, such as specific imaging modalities beyond routine radiography or histological examination, could be considered a limitation. Another limitation is the lack of a comparator group of elderly residents not receiving BP or Dmab. This absence prevents a direct assessment of the causal relationship between antiresorptive agent use and MRONJ development in this specific population. Future studies incorporating a control group would be beneficial to establish this association more definitively. Finally, the fact that this report focuses on special nursing homes warrants consideration as a potential confounding factor. Variations in resident characteristics (e.g., prevalence of dementia, level of care dependency), oral hygiene practices, and access to dental care across different types of elderly care facilities could influence both BP/Dmab usage and MRONJ incidence. Future research should explore these differences by including a more diverse range of facility types and potentially performing stratified analyses based on facility characteristics.

## Conclusions

In these elderly facilities, 9.8% of residents used BPs or Dmab, with 15.6% receiving injectable formulations. One MRONJ case occurred with injectable BP use, suggesting the potential for sporadic MRONJ in elderly care. This study provides preliminary data on the prevalence of BP/Dmab use and the occurrence of MRONJ in elderly care facilities, emphasizing the potential risks linked to injectable formulations. The findings underscore the importance of heightened awareness and interprofessional collaboration among healthcare professionals in these settings for the early detection and prevention of MRONJ. Future research with larger, multi-center studies is necessary to validate these findings and to develop evidence-based preventive strategies tailored to this vulnerable population.
